# The Neuroprotective Potentials of Dual GIP/GLP1‐RA (Tirzepatide): From Preclinical Experiments to Clinical Trials: A Scoping Review

**DOI:** 10.1002/brb3.71702

**Published:** 2026-08-17

**Authors:** Yousef Hawas, Mohamed Abouzid, Ahmed Farid Gadelmawla, Dalia Kamal Ewis, Abdallah Khatatbeh, Yasmin Negida, Ahmed Negida

**Affiliations:** ^1^ Faculty of Medicine Tanta University Tanta Egypt; ^2^ Medical Research Group of Egypt Arlington Massachusetts USA; ^3^ Department of Physical Pharmacy and Pharmacokinetics Poznan University of Medical Sciences Poznan Poland; ^4^ Faculty of Medicine Menoufia University Shebin El‐Kom Egypt; ^5^ Faculty of Medicine Beni Suef University Beni Suef Egypt; ^6^ Al‐Hussein Medical Center Jordanian Royal Medical Services Amman Jordan; ^7^ Faculty of Medicine Zagazig University Zagazig Egypt; ^8^ Department of Neurology Virginia Commonwealth University Richmond Virginia USA

**Keywords:** Alzheimer, cerebrovascular, dementia, glucose‐dependent insulinotropic polypeptide (GIP)/glucagon‐like peptide 1 (GLP‐1), neuroprotection, Parkinson, tirzepatide

## Abstract

**Background:**

Tirzepatide (TZP), the first approved dual glucagon‐like peptide 1 (GLP‐1)/glucose‐dependent insulinotropic polypeptide (GIP) agonist, has shown neuroprotective effects in preclinical models and early observational studies. This review maps and appraises current preclinical and clinical evidence on TZP's potential neuroprotective effects in neurodegenerative diseases (Alzheimer's and Parkinson's disease), neuroinflammation, cerebrovascular outcomes, neurovestibular disorders, brain tumor response, and idiopathic intracranial hypertension.

**Methods:**

PubMed, Scopus, Web of Science, Embase, and Elton B. Stephens Company (EBSCO) were searched through September 30, 2025. Eligible sources included original preclinical or clinical studies reporting neurological or neuroprotective effects of TZP. Quality was assessed using SYstematic Review Centre for Laboratory animal Experimentation (SYRCLE) and Collaborative Approach to Meta‐Analysis and Review of Animal Data from Experimental Studies (CAMARADE) for in vivo studies, toxicological data reliability assessment tool (ToxRTool) for in vitro, Newcastle‐Ottawa scale (NOS) for cohorts, and Joanna Briggs Institute (JBI) for case reports.

**Results:**

Twenty‐two studies met inclusion criteria as follows: 11 preclinical investigations, 3 retrospective cohorts, and 8 case reports. Preclinical studies demonstrated mitochondrial stabilization (adenosine triphosphate [ATP] restoration; PINK1/Parkin), anti‐oxidative and anti‐inflammatory signaling, synaptic support (PSD‐95/synaptophysin), and blood–brain barrier reinforcement (claudin‐1), alongside behavioral improvements. Retrospective cohorts reported reduced cerebrovascular and neurodegenerative disease incidence, papilledema, and headaches, though one cohort identified higher risk of central vertigo and vestibular neuronitis. Case reports documented compression neuropathies, autoimmune encephalitis, hypoglycemia‐related seizures, psychiatric reactions, and rhabdomyolysis, most resolving with dose adjustment or discontinuation. Risk‐of‐bias appraisal revealed frequent “unclear” ratings in animal studies, “reliable with restrictions” in vitro, and high NOS scores for cohorts.

**Conclusions:**

TZP exhibits biologically plausible neuroprotective mechanisms preclinically. Prospective trials with standardized neurological endpoints, active‐comparator designs, and structured safety monitoring are warranted.

## Graphical abstract

The graphical abstract summarizes the current evidence on the potential neuroprotective effects of tirzepatide (TZP), a dual GIP/GLP‐1 receptor agonist, across mechanistic, preclinical, and clinical domains. Mechanistically, TZP is proposed to exert direct neural effects, preserve mitochondrial function, reduce apoptosis, oxidative stress, and neuroinflammation, and activate neuroprotective pathways including PI3K/Akt signaling and BDNF. Among the 22 studies mapped in this PRISMA‐ScR‐guided review, nine preclinical studies demonstrated improvements in motor and cognitive outcomes in experimental models of Parkinson's and Alzheimer's diseases, while three human cohort/database studies suggested associations with a reduced risk of dementia and stroke. Neurological adverse events were reported mainly in case reports and were predominantly associated with rapid weight loss or hypoglycemia, with most being reversible.

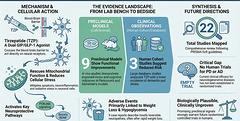

**Abbreviations**
AD: Alzheimer's disease
AMPK: AMP‐activated protein kinase
ATP: adenosine triphosphate
Aβ: amyloid beta
BBB: blood–brain barrier
BDNF: brain‐derived neurotrophic factor
CAMARADES: Collaborative Approach to Meta‐Analysis and Review of Animal Data from Experimental Studies
CREB: cAMP response element‐binding protein
EBSCO: Elton B. Stephens Company (online research database provider)
EHR: electronic health record
FDA: food and drug administration
GIP/GLP‐1 RA: dual glucose‐dependent insulinotropic polypeptide/glucagon‐like peptide‐1 receptor agonist
GIT: gastrointestinal tract
HbA1c: glycated hemoglobin A1c
IIH: idiopathic intracranial hypertension
JBI: Joanna Briggs Institute
NLRP3: NOD‐like receptor family pyrin domain containing 3
NOS: Newcastle–Ottawa Scale
OGD/R: oxygen–glucose deprivation/reoxygenation
PD: Parkinson's disease
PI3K: phosphatidylinositol‐3 kinase
PKA: protein kinase A
PRISMA‐ScR: Preferred Reporting Items for Systematic Reviews and Meta‐Analyses extension for Scoping Reviews
PROSPERO: international prospective register of systematic reviews
RCT: randomized controlled trial
ROS: reactive oxygen species
SIRT3: sirtuin 3
SYRCLE: Systematic Review Centre for Laboratory animal Experimentation
T2DM: Type 2 diabetes mellitus
TEER: transendothelial electrical resistance
ToxRTool: toxicological data reliability assessment tool
TZP: tirzepatide

## 1 Introduction

Glucose‐dependent insulinotropic polypeptide (GIP) and glucagon‐like peptide 1 (GLP‐1) were discovered in the 20th century and are involved in glucose level regulation (Alsalim et al. 2022). GIP was discovered first, and it is secreted from K cells in the gastrointestinal tract (GIT), whereas GLP‐1 was discovered later and is secreted from L cells. Both incretin hormones are secreted in response to food rich in carbohydrates and fat, and their levels increase postprandially (Zheng et al. 2024). They increase insulin secretion, and their effect was tested on animals with impaired insulin receptor sensitivity and secretion. They showed good efficacy as hypoglycemic agents, but with very short half‐lives. The first GLP‐1 agonist, exendin‐4, and other GLP‐1 receptor agonists, such as semaglutide and liraglutide, were developed and approved by the food and drug administration (FDA) to treat Type 2 diabetes mellitus (T2DM), besides lifestyle modification, with a longer half‐life. They stimulate beta cells’ growth and secretion of insulin and decrease glucagon secretion (Buteau 2008).

GLP‐1 receptors are widely distributed in the brain, neurons, hippocampus, and pyramidal cells, and GLP‐1 agonists can penetrate the blood–brain barrier (BBB) with sufficient concentrations, which are deficient in neurotrophic growth factors. Due to these properties of GLP‐1R agonists and the above‐mentioned similarities and association between DM and neurological diseases, these agonists were investigated in clinical and preclinical research to explore their neuroprotective action (Yang et al. 2025). In preclinical studies, GLP‐1 showed neuroprotective activity via anti‐apoptotic and anti‐inflammatory effects and activation of PKA (protein kinase A) and PI3K (phosphatidylinositol‐3) kinase pathway (Reich and Hölscher 2022). Moreover, GIP receptor agonists reduce amyloid plaques, α‐synuclein aggregation, chronic inflammation, oxidative stress, and mitochondrial dysfunction while boosting synaptic plasticity, brain‐derived neurotrophic factor (BDNF) levels, neurogenesis, and dopamine neuron survival. Protease‐resistant GIP analogs cross the BBB, normalizing motor function in Parkinson's disease (PD) models and rescuing memory in Alzheimer's disease (AD) models (Ko et al. 2024; Ji et al. 2016).

Tirzepatide (TZP) is the first drug with dual action on GLP‐1 and GIP receptors, leveraging the synergistic benefits of acting on two receptors. TZP has a long half‐life due to albumin binding. It's injected subcutaneously once weekly with a starting dose of 2.5 mg and titrated to 5 mg over 4 weeks with a maximum dose of 15 mg. TZP has a weaker effect on GLP‐1 than other GLP‐1RAs but similar potency as GIP agonists. Mechanistically, TZP is an imbalanced, biased dual agonist: it engages the GIP receptor more completely than the GLP‐1 receptor and, at the GLP‐1 receptor, favors cAMP generation over β‐arrestin recruitment (Willard et al. 2020). This pharmacological asymmetry is relevant when inferring central effects, because dual agonism cannot be assumed to be simply additive over GLP‐1 receptor monoagonism. TZP was approved by the FDA in 2022 for T2DM (Nauck and D‘Alessio 2022). It was subsequently approved for chronic weight management and, in 2024, for moderate‐to‐severe obstructive sleep apnea in adults with obesity (Malhotra et al. 2024).

TZP was investigated in clinical trials for treating T2DM and obesity and showed a significant reduction in blood glucose levels, glycated hemoglobin A1c (HbA1c), a decrease in body weight, and waist circumference. TZP exhibited non‐inferior and even superior efficacy to GLP‐1R agonists, with a comparable safety profile; the most frequent adverse events were gastrointestinal (Sinha et al. 2023). Moreover, TZP is being evaluated for heart failure, cardiovascular protective effects, and fatty liver (Syed 2022). Given the history of GLP‐1RAs as neuroprotective agents, but not robust efficacy, and superiority of TZP as a dual agonist, and proven efficacy and safety compared to other GLP‐1RAs in clinical trials, it has been tested in preclinical and clinical trials as a neuroprotective agent (Olukorode et al. 2024). Recent studies featured the neuroprotective effect of TZP in neurological diseases such as AD. Reduced inflammation, enhanced insulin signaling, and improved cognition were proposed as mechanisms of action (Alhowail et al. 2025). These mechanisms are similar between neurological disorders; recently published studies have proposed TZP as a neuroprotective agent.

Although GLP1‐RAs have been studied in neurological disorders, TZP remains relatively underexplored. GIP receptor agonists also showed neuroprotective effects (Ji et al. 2016; Zhang and Hölscher 2020; Zhang et al. 2023). Our hypothesis assumes that dual GIP and GLP‐1‐RA confer potentially neuroprotective effects relative to traditional GLP‐1 RA, representing a novel and relevant area for exploration. We conducted this scoping review to systematically assess the clinical and preclinical evidence, synthesize qualitative evidence, and critically appraise the published studies. We investigated the current evidence of TZP in terms of different neurological conditions, including cerebrovascular diseases, neurodegenerative diseases such as Alzheimer's, PD, idiopathic intracranial hypertension (IIH), vestibular diseases, and any other neurological conditions reported in the literature. This will guide future studies to consider the gaps and help develop and improve further investigation of dual GIP/GLP‐1 receptor agonists, particularly TZP.

## 2 Methods

### 2.1 Protocol and Registration

This scoping review followed the guidelines described by Arksey and O'Malley and the Preferred Reporting Items for Systematic Reviews and Meta‐Analyses extension for Scoping Reviews (PRISMA‐ScR) guidelines (Moher et al. 2009; Arksey and O'Malley 2005). This review was registered in the PROSPERO (international prospective register of systematic reviews) (ID: CRD420251181376).

### 2.2 Search Strategy

PubMed, Scopus, Web of Science, Embase, and Elton B. Stephens Company (EBSCO) databases were searched up to September 30, 2025. We focused on using TZP‐related terms exclusively and did not include any neurological terms to ensure a comprehensive search (Tirzepatide OR LY3298176 OR Zepbound OR Mounjaro OR GIP/GLP‐1 OR BG‐121 OR GLP‐1RA OR GIP/GLP‐1 RA). No filters or language restrictions were applied. The studies’ references were also screened to ensure all relevant articles were covered.

### 2.3 Inclusion Criteria

The objective of this scoping review was to summarize the current evidence of the neuroprotective activities of TZP from preclinical to clinical studies. Thus, our review included all original studies reporting on the neuroprotective effects of TZP. No restrictions were placed on the studied disease, population, or outcomes. The inclusion criteria covered (i) randomized controlled trials (RCTs), observational studies, case reports, and case series; (ii) animal studies; and (iii) in vitro studies. Reviews, editorials, and studies on non‐neurological diseases were excluded. Abstracts without full original articles, whether cohorts, in vivo, or in vitro, were excluded. Moreover, studies that included GLP1 agonists, such as semaglutide, without presenting a separate analysis for TZP were excluded to ensure presenting pure evidence of TZP without overlapping with other medications. However, those studies were mentioned or discussed in the text but not extracted or underwent quality assessment.

### 2.4 Study Selection and Data Extraction

Two authors (D.K.E. and A.K.) independently screened the titles and abstracts of the citations. Then, a third author (Y.H.) retrieved and screened the full text of the identified studies to make the final decision. After identifying the included studies, two authors (D.K.E and A.K.) extracted the data using an online data extraction form. For cohorts, the extracted data included information such as country, population, sample size, eligibility criteria, safety and efficacy outcomes, main conclusion, and limitations. For animal studies, the extracted data encompassed the animal model, sample size, timing of administration, duration of study, follow‐up timepoints, behavioral, mechanistic, molecular and histological outcomes, findings, safety, main findings, and limitations. Any discrepancies in the screening process or data extraction were resolved through discussion with a third author (Y.H.). For in vitro studies, the following items were extracted: country, cell type and source, purpose of model (e.g., neurodegeneration under hyperglycemic stress), glucose condition, TZP source and solvent, exposure duration, additional interventions or treatments, replicates and sample size, assays used, primary outcomes, mechanistic findings, main conclusion, and limitations. For case reports, the following data were extracted: country, patient demographics, primary disease, main problem, clinical presentation and diagnosis, timing after TZP given, dosage when drug reaction occurs, first time intervention measures, investigations, management, improvement after managing, return time, drug rechallenge, combined medication, and brief sJummary.

### 2.5 Quality Assessment

We followed the toxicological data reliability assessment tool (ToxRTool) to assess the quality of the in vitro studies. It is divided into five main domains that assess documentation of substance used, test system, reporting documentation, methodology, and plausibility (Schneider et al. 2009). For in vivo studies, SYstematic Review Centre for Laboratory animal Experimentation (SYRCLE's) checklist was used to assess their quality. SYRCLE's Risk of Bias Tool is adapted from the Cochrane risk‐of‐bias instrument. It covers domains, such as selection, performance, detection, attrition, and reporting bias (Hooijmans et al. 2014). Moreover, the Collaborative Approach to Meta‐Analysis and Review of Animal Data from Experimental Studies (CAMARADE) tool assesses the quality of animal studies by providing a total score that indicates the overall quality of the study (Hamad et al. 2024). Newcastle‐Ottawa scale (NOS) was used to assess cohort studies. It is based on a star rating system based on selection, comparability, and outcome/exposure assessment (Wells et al. 2011). Case reports were assessed using the Joanna Briggs Institute (JBI) tool. It addresses key domains such as the clarity of patient history, clinical condition, diagnostic tests, interventions, and outcomes (JBI 2025).

### 2.6 Analysis

Due to heterogeneity in the included studies regarding populations and outcomes, we conducted a qualitative data analysis following the recommended methodology for qualitative reviews outlined in the Cochrane Handbook. We categorized the manuscript into two groups as follows: (i) preclinical and (ii) clinical. For the preclinical studies (animal and in vitro studies), we tabled the following information: (i) animal model utilized (e.g., mouse and pig); (ii) sample size; (iii) age, sex, and weight of the animals; (iv) outcome measures assessed; (v) main findings; and (vi) study quality evaluation. Regarding the clinical studies, we gathered the following details: (i) study design; (ii) characteristics of the study population (i.e., age and disease condition); (iii) outcome measures examined; (iv) effects of TZP on the outcome measures; and (v) evaluation of study quality using the aforementioned criteria.

## 3 Results

### 3.1 Selection of Sources of Evidence

**FIGURE 1:**
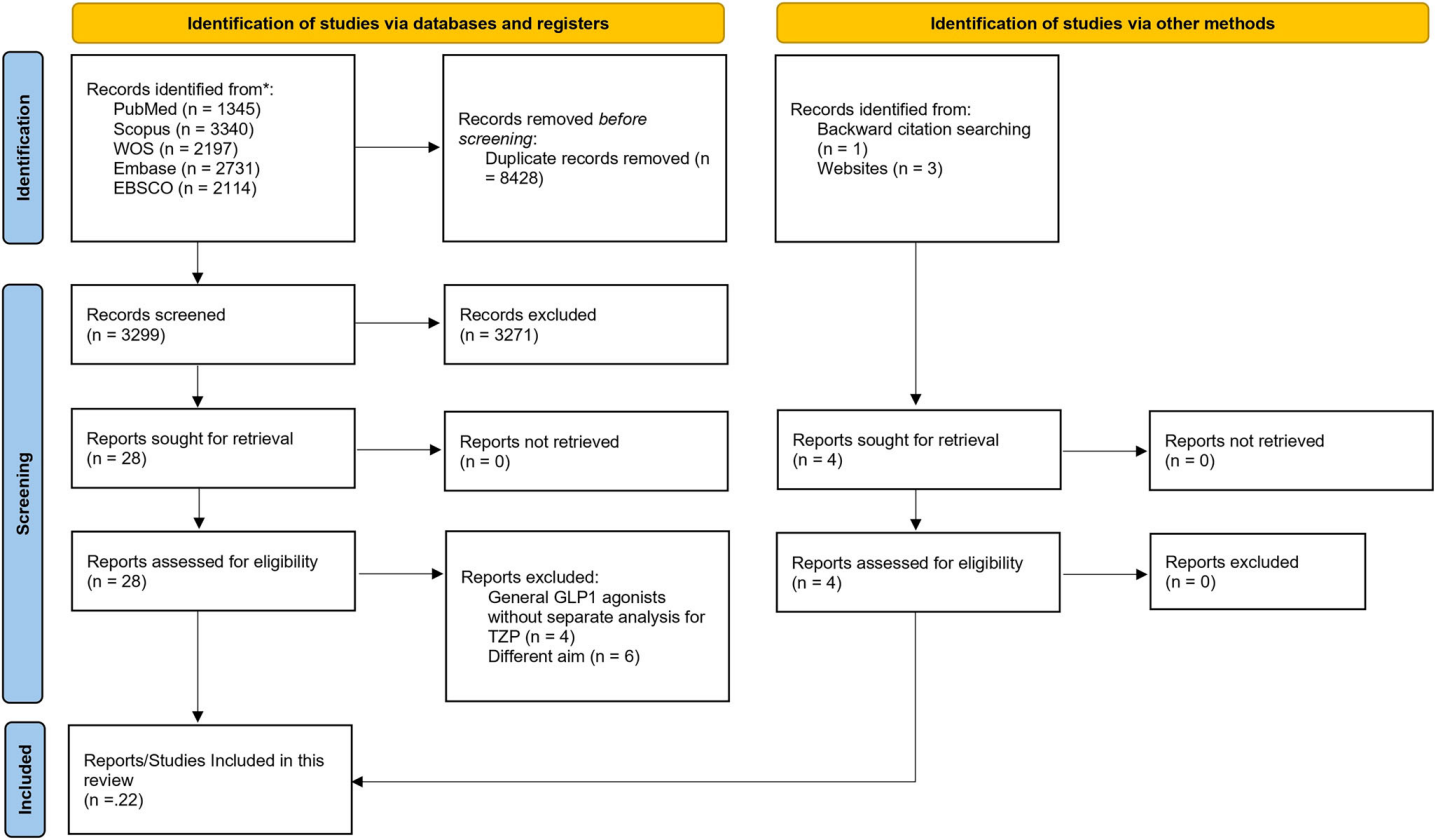
PRISMA 2020 flow diagram. EBSCO, Elton B. Stephens Company (online research database provider); GLP‐1, glucagon‐like peptide 1; TZP, tirzepatide.

Following PRISMA‐ScR 2020, we identified 11,727 records from PubMed, Scopus, WOS, Embase, and EBSCO databases and four records from other sources (backward citation searching:1; manual searching on websites: 3). After removal of 8428 duplicates, 3299 records were screened on the basis of title and abstract, and 3271 were excluded. We assessed 28 database reports and four additional reports in full text; 10 were excluded (general GLP‐1 agonists without separate TZP analysis, *n* = 4; different aim, *n* = 6). In total, 22 studies were included (Figure 1).

### 3.2 Characteristics of Sources of Evidence

We reviewed 22 sources as follows: 11 preclinical publications, 3 retrospective cohort studies, and 8 case reports describing nine individual cases. Within the preclinical body, we charted 14 parts: 5 in vitro and 9 in vivo. Three publications were mixed in vivo and in vitro; they were counted once in the PRISMA total but charted twice, once per part, for characteristics and outcome denominators. The three clinical studies were retrospective propensity score‐matched cohorts from TriNetX database.

### 3.3 Preclinical Studies

#### 3.3.1 In Vitro Studies

Five in vitro reports were included. Two were purely in vitro (Wang, Chen, et al. 2025; Fontanella et al. 2024), and three were in vitro reports within mixed in vivo and in vitro studies (Tian et al. 2025; Yang et al. 2024; Wang, Wang, et al. 2025). Across neuronal systems, astrocytes, brain endothelial cells, and glioblastoma lines, TZP most modulated cellular stress biology, typically improving mitochondrial and bioenergetic readouts (e.g., mitochondrial membrane potential and adenosine triphosphate [ATP]‐related indices) and reducing oxidative stress and apoptosis in non‐tumor neural models, while showing context‐dependent pro‐ferroptotic effects in glioblastoma lines (Table 1).

**TABLE 1:** Summary TZP of in vitro studies.

Study ID	Country	Cell type and source	Purpose of model (e.g.)	Glucose condition	TZP source and solvent	Exposure duration	Additional interventions or treatments	Replicates/Sample size	Assays used	Primary outcomes	Mechanistic findings	Main conclusion	Limitations
Tian 2025—in vitro part (Tian et al. 2025)	China	Human neuroblastoma SY5Y cells (ShareBio, Shanghai, China)	To mimic Parkinson's disease neurodegeneration in vitro using MPP^+^ (toxic metabolite of MPTP) and evaluate TZP's effects on mitochondrial homeostasis	Standard DMEM with 10% FBS (not glucose‐modified)	TZP 1 µM (source: not explicitly stated for in vitro, but likely same supplier—Selleck P1206; solvent not specified, standard cell culture medium vehicle)	Not explicitly stated; assays conducted after treatment period (standard in vitro PD assays—typically 24–48 h; authors did not clarify exact timing)	‐ MPP^+^ (1 mM) to induce PD‐like injury—Chloroquine (20 µM, lysosomal inhibitor)—3‐methyladenine (10 mM, autophagy inhibitor)	*n* = 6 per group	‐ Mitochondrial membrane potential (MMP) assay—ATP quantification assay—NAD+/NADH ratio assay	TZP rescued MPP^+^‐induced decline in MMP, ATP, and NAD+/NADH ratio; protective effects abolished when autophagy/lysosomal pathways were inhibited	TZP maintains mitochondrial homeostasis via mitophagy; its protective effect depends on intact autophagy/lysosomal function	TZP ameliorates MPP^+^‐induced mitochondrial dysfunction in SY5Y cells by promoting mitophagy and maintaining energy homeostasis	Exact exposure duration and solvent not specified; findings limited to a single cell line; mechanistic claims need genetic validation (e.g., siRNA/CRISPR for Pink1, Parkin, Drp1)
Jiangtao Wang, Chen, et al. (2025)	China	Human glioblastoma cell lines: U87, LN229, T98G	Assess TZP anti‐glioblastoma activity via ferroptosis induction	Standard culture media; no high‐glucose stress reported	200 to 400 nM TZP (solvent not specified)	24 h	Ferrostatin‐1 (5 µM), SOX2 overexpression, GIPR antagonist (GIP (3–30)), and GLP‐1R antagonist (Exendin (9–39))	Not specified	ROS (DCFH‐DA), MDA, Fe^2+^, GSH/GSSG, SOD, qPCR, Western blot, migration assays	Increased ROS/MDA/Fe^2+^; decreased SOD and GSH/GSSG; decreased GPX4, increased ACSL4; decreased SOX2 and SLC7A11; effects rescued by Fer‐1 or SOX2 overexpression	TZP triggers ferroptosis through SOX2/SLC7A11 suppression; GIPR partly involved, GLP‐1R minimal role	TZP induces ferroptosis in glioblastoma cells via SOX2/SLC7A11 axis. May be a therapeutic candidate for glioblastoma	High in vitro concentrations; lack of noncancerous control cells; in vivo validation needed
Duozi Wang 2025—in vitro part (Wang, Wang, et al. 2025)	China	Human brain microvascular endothelial cells (HBMVECs, ATCC, USA)	Ischemia‐like injury via oxygen‐glucose deprivation/reperfusion (OGD/R) to model stroke‐induced BBB disruption	Glucose‐free medium during OGD (10 h) followed by reperfusion with normal medium (24 h)	TZP (Eli Lilly); dissolved in DMSO (0.1%)	24 h reperfusion phase post‐OGD with TZP (50 or 100 nM)	C/EBP‐α silencing using Ad‐viral shRNA (48 h before OGD/R)	Triplicates per condition; CV <10% for permeability assays	TEER, FITC‐dextran permeability, Western blot (Claudin‐1, C/EBP‐α), RT‐PCR, Immunostaining	Endothelial permeability, TEER values, Claudin‐1, and C/EBP‐α expression	TZP restored Claudin‐1 expression and improved TEER by activating C/EBP‐α; knockdown abolished protective effect	TZP protects BBB endothelial integrity under ischemia‐like stress via Claudin‐1 upregulation and C/EBP‐α signaling	Limited to in vitro BBB model; results may not fully replicate in vivo complexity
Yang 2024—in vitro part (Yang et al. 2024)	China	Primary cortical neurons; N2a neuroblastoma cells; C8‐D1A astrocytes	Investigate TZP protective effects against Aβ‐induced neuronal injury	Normal glucose; with Aβ treatment to simulate AD pathology	10 nM TZP (solvent not reported)	24 h co‐incubation with Aβ	Aβ oligomers exposure; GLP‐1R siRNA knockdown	Experiments repeated 3 times	Hoechst staining (apoptosis), ATP, ROS, mitochondrial membrane potential (MMP), Western blot (Tom20, GLP‐1R)	Decrease apoptosis; Increased ATP; Decreased ROS; preserved MMP; GLP‐1R knockdown abolished protective effects	GLP‐1R dependent, mitochondrial protection (Tom20), improved bioenergetics	TZP protects neurons/astrocytes from Aβ‐induced apoptosis and mitochondrial dysfunction	In vitro only; single concentration; no long‐term assays
Fontanella et al. (2024)	Italy	SH‐SY5Y human neuroblastoma (DSMZ ACC‐209)	Model neurodegeneration and insulin resistance under high glucose; test TZP neuroprotection	NG 25 mM vs. HG 150 mM for 7 days	TZP (Selleckchem LY3298176, cat. P1206), 0.2 µM; media changed every 48 h	7 days	None beyond NG/HG ± TZP	Experiments repeated ≥3 times; qPCR run in duplicates; densitometry from three experiments	Ki‐67 flow cytometry, Western blot, RT‐qPCR, DNA methylation pyrosequencing, miRNA qPCR; in silico IPA pathway analysis	Increased CREB/BDNF, Increased pAkt, Increased MAP2/GAP43/AGBL4; Decreased BAX/Bcl‐2 ratio; Increased GLUT3/GLUT4 and SORBS1; TIR prevented HG‐induced hypermethylation of CREB/BDNF; lower miR‐34a, elevated miR‐212/29c; HG effects reversed	Activation of pAkt, CREB, lead to BDNF signaling; improved neuronal glucose transport (GLUTs/SORBS1); epigenetic modulation of CREB/BDNF and miRNAs	TZP ameliorates HG‐induced neurodegeneration and insulin resistance in SH‐SY5Y through multiple molecular pathways	In vitro findings; authors note need for clinical validation

Abbreviations: µM, micromolar (unit of concentration); ACSL4, Acyl‐CoA synthetase long chain family member 4; AD, Alzheimer's disease; Ad‐viral, adenoviral (related to adenovirus vectors); AGBL4, ATP/GTP binding protein like 4; ATP, adenosine triphosphate; Aβ, amyloid beta; BAX, Bcl‐2‐associated X protein; BBB, blood–brain barrier; Bcl‐2, B‐cell lymphoma 2; BDNF, brain‐derived neurotrophic factor; C/EBP‐α, CCAAT/enhancer binding protein alpha; CREB, cAMP response element‐binding protein; CRISPR, clustered regularly interspaced short palindromic repeats; CV, coefficient of variation; DCFH‐DA, 2′,7′‐dichlorodihydrofluorescein diacetate (ROS detection probe); DMEM, Dulbecco's Modified Eagle Medium; DMSO, dimethyl sulfoxide; Drp1, dynamin‐related protein 1; DSMZ, German Collection of Microorganisms and Cell Cultures; FBS, fetal bovine serum; Fe^2+^, ferrous ion; Fer‐1, ferrostatin‐1 (ferroptosis inhibitor); FITC, fluorescein Isothiocyanate; GAP43, growth associated protein 43; GIPR, gastric inhibitory polypeptide receptor; GLP‐1R siRNA, small interfering RNA targeting GLP‐1 receptor; GLP‐1R, glucagon‐like peptide‐1 receptor; GLUT3/GLUT4, glucose transporter Types 3 and 4; GPX4, glutathione peroxidase 4; GSH/GSSG, reduced/oxidized glutathione; HBMVECs, human brain microvascular endothelial cells; HG, high glucose; IPA, ingenuity pathway analysis; LN229; MAP2, microtubule‐associated protein 2; MDA, malondialdehyde; miRNA, microRNA; MMP, mitochondrial membrane potential; MPP^+^, 1‐methyl‐4‐phenylpyridinium (toxic metabolite of MPTP); MPTP, 1‐Methyl‐4‐phenyl‐1,2,3,6‐tetrahydropyridine (induces Parkinson's‐like symptoms); NAD+/NADH, nicotinamide adenine dinucleotide (oxidized/reduced forms); NG, normal glucose; nM, nanomolar (unit of concentration); OGD/R, oxygen–glucose deprivation/reperfusion; Parkin, Parkin RBR E3 ubiquitin protein ligase; PD, Parkinson's disease; Pink1, PTEN‐induced putative kinase 1; qPCR, quantitative polymerase chain reaction; ROS, reactive oxygen species; RT‐PCR, reverse transcription polymerase chain reaction; Selleckchem, supplier of biochemical reagents; shRNA, short hairpin RNA; SH‐SY5Y, human neuroblastoma cell line (DSMZ ACC‐209); siRNA, small interfering RNA; SLC7A11, solute carrier family 7 member 11; SOD, superoxide dismutase; SORBS1, sorbin and SH3 domain containing 1; SOX2, SRY‐box transcription factor 2; SY5Y, human neuroblastoma cell line; T98G, human glioblastoma cell lines; TEER, transendothelial electrical resistance; TIR: TZP; Tom20, translocase of outer mitochondrial membrane 20; TZP, tirzepatide; TZP: TZP; U87.

In dopaminergic toxin injury (SH‐SY5Y + MPP^+^) (Tian et al. 2025), TZP reduced apoptosis and oxidative stress and improved mitochondrial indices, with loss of benefit when autophagy/lysosomal pathways were inhibited, supporting a mitophagy‐linked mechanism. In amyloid‐β injury models (primary neurons/N2a and astrocytes) (Yang et al. 2024), TZP reduced apoptosis, lowered reactive oxygen species (ROS), and preserved mitochondrial function; GLP‐1 receptor knockdown abolished these effects, supporting receptor‐dependent protection. At the neurovascular interface (HBMVECs under oxygen–glucose deprivation/reoxygenation [OGD/R]) (Wang, Wang, et al. 2025), TZP improved barrier integrity (higher transendothelial electrical resistance [TEER], lower permeability) through C/EBP‐α–linked restoration of Claudin‐1; C/EBP‐α silencing removed the effect. Under high‐glucose neuronal stress (Fontanella et al. 2024), TZP increased p‐Akt and cAMP response element‐binding protein (CREB)/BDNF signaling, improved synaptic markers, and prevented stress‐associated CREB/BDNF hypermethylation with accompanying miRNA shifts. In glioblastoma cell lines (Wang, Chen, et al. 2025), TZP shifted ferroptosis‐related biology via a SOX2/SLC7A11 axis, with partial GIPR involvement and limited GLP‐1R contribution.

#### 3.3.2 In Vivo Findings

Nine in vivo reports were included as follows: six reports of in vivo studies (Delvadia et al. 2025; Misra et al. 2025; Hassan et al. 2024; Forny Germano et al. 2024; Guo et al. 2023; Ma et al. 2025) and three reports from mixed in vivo and in vitro studies (Tian et al. 2025; Yang et al. 2024; Wang, Wang, et al. 2025). Across toxin Parkinsonism, Alzheimer‐type transgenic models, metabolic–cognitive impairment, diabetes‐related cognitive deficit, and drug‐induced neurotoxicity, TZP generally improved behavioral outcomes and/or preserved neuronal and synaptic integrity alongside reductions in neuroinflammation and oxidative stress (Table 2). The formulation, dose, route, and timing of administration relative to injury (pre‐ versus post‐insult) for every included study are detailed in Tables 1 and 2. Notably, most in vivo studies administered TZP after toxin exposure or injury induction, modeling a therapeutic rather than a prophylactic paradigm.

**TABLE 2:** Summary of tirzepatide (TZP) effect in In vivo related studies.

Study ID	Country	Species and strain (C57BL/6 mice, etc)	Age, sex, and weight	Model type (Diabetes type II and APP/PS1 Alzheimer's model)	Sample size (number of animals per group)	TZP dose, formulation, route	Timing of administration (before or after the induction of injury)	Duration of study	Follow‐up timepoints	Behavioral outcomes (water maze, Grip strength, rotarod, etc)	Histological outcomes (infarct volume, Neuronal survival, etc)	Molecular outcomes (cytokines, oxidative stress, apoptosis markers, etc)	Mechanistic outcomes (signaling pathway modulated)	Safety (mortality, weight changes, etc)	Main findings (summary of neuroprotective effects)	Limitations
Tian 2025—in vivo (Tian et al. 2025)	China	Male C57BL/6 mice	8 weeks old, male, 22 ± 2 g	MPTP‐induced subacute Parkinson's disease mouse model	*n* = 9 per group (Vehicle, MPTP, TZP, Semaglutide, Levodopa); additional groups with Drp1 inhibitor and mitophagy activator also *n* = 9	10 nmol/kg, intraperitoneal (i.p.), Selleck P1206	After induction of PD by MPTP (30 mg/kg i.p. × 5 days)	4 weeks treatment after MPTP induction	After 4 weeks of treatment then behavioral tests, histology, molecular assays	TZP improved locomotor activity (increased hind‐leg stands, more square crossings, improved traction scores) compared to untreated MPTP mice; effects comparable to semaglutide and levodopa	Preserved tyrosine hydroxylase (TH)‐positive neurons in substantia nigra; reduced dopaminergic neuron loss compared to MPTP group	Restored ATP content in brain tissue; reduced Drp1 expression; reversed MPTP‐induced suppression of Pink1, Parkin, and p62 expression	Protective effects mediated by regulating mitochondrial dynamics: inhibition of Drp1‐mediated mitochondrial fission, promotion of Pink1‐Parkin‐dependent mitophagy, improved mitochondrial ultrastructure	No mortality reported; model chosen (subacute MPTP) had lower mortality than acute regimens; no specific adverse effects mentioned	TZP protects dopaminergic neurons and motor function in PD mice by restoring mitochondrial energy homeostasis and enhancing mitophagy. Effects comparable to semaglutide and levodopa, despite using one‐third the dose of semaglutide	Need for further studies on optimal dosing across acute/chronic PD models; lack of genetic validation (e.g., CRISPR) to confirm role of Drp1‐Pink1‐Parkin pathway; limited toxicity/pharmacokinetic evaluation
Duozi Wang 2025—in vivo (Wang, Wang, et al. 2025)	China	Male ICR mice (23–25 g)	Male, 23–25 g	Transient MCAO (right MCA occlusion 1 h followed by reperfusion) ischemic stroke model	Typically *n* = 6 per group for BBB permeability (^14^C‐sucrose) and *K*_p, brain_ and P‐gp sub‐studies	10 nmol/kg, intraperitoneal, weekly for 8 weeks; vehicle 0.1 mL saline intraperitoneal	Start 24 h post‐MCAO	8 weeks of treatment	Outcomes assessed after the treatment course; *K*_p, brain_ measured 24 h after final dose; a mechanistic arm reports neuro scores at 3 days post‐MCAO	Neurological score (Longa 0–4): MCAO from 3.5–3.6 to 1.6–1.4 with TZP (improved deficits)	TTC infarct volume: MCAO from 38.52% ± 4.2% to 18.72% ± 3.1% with TZP (reduced infarct)	BBB permeability (^14^C‐sucrose Ki): Sham 1.00 ± 0.028 increased to MCAO 2.582 ± 0.156 and decreased to 1.447 ± 0.103 with TZP (reduced leak). Tight‐junction proteins restored (Claudin‐1 increased; also ZO‐1/occludin reported as restored). *K*_p, brain_ for TZP 0.12–0.14 (higher than published liraglutide approximately 0.03)	C/EBP‐α signaling was activated with tirzepatide. Silencing or knockout of C/EBP‐α abolished tirzepatide's improvements in neurological score, blood–brain barrier permeability, and Claudin‐1 expression, which supports the role of the C/EBP‐α and Claudin‐1 axis in BBB r repair	No overt behavioral toxicity reported; mortality/adverse effects NR. P‐gp inhibition (verapamil) did not significantly change plasma, brain, or *K*_p, brain_ of TZP	Post‐stroke tirzepatide (10 nmol/kg intraperitoneally weekly) improved neurological deficits, reduced infarct size, reduced BBB leak, and restored tight junction proteins by activating the C/EBP‐α and Claudin‐1 pathway. Tirzepatide shows moderate central nervous system penetration (*K*_p, brain_ approximately 0.12–0.14)	^14^C‐sucrose reflects paracellular leak (not active transport); possible LC–MS/MS matrix effects; limited time‐point sampling; long‐term/CNS safety not defined; efflux transporters beyond P‐gp (e.g., BCRP) not assessed; direct mechanism of BBB crossing not proven
Delvadia et al. (2025)	India	Wistar rats	Male, 6–8 weeks, 200–225 g	Rotenone (2 mg/kg s.c.) induced Parkinson's disease model	6 groups; *n* = 10/group (normal; rotenone; exendin‐4; TZP 100 nmol/kg; rotenone + TZP 50 nmol/kg; rotenone + TZP 100 nmol/kg)	TZP (50 and 100 nmol/kg, s.c.), dissolved in phosphate‐buffered saline once a day for 28 days	TZP/exendin‐4 given 2 h after rotenone each day	28 days	End of Week 4 (behavior)	Locomotor activity (infrared actimeter), Catalepsy test, Rotarod latency	α‐Synuclein and pSer129 α syn expression (WB); general histopathology	Increased striatal dopamine (HPLC); reduced TNF‐α and IL‐6 (ELISA); reduced MDA; increased GSH, SOD; GSSG; protein assays	Anti‐inflammatory and anti‐oxidative effects; reduction of α‐syn aggregation	Not reported	Dose‐dependent neuroprotection; 100 nmol/kg most effective vs. rotenone to improve motor function, raise DA, and suppress inflammation/oxidative stress	Toxin PD model; EHR on group count (text reports *n* = 10/group); short duration
Misra et al. (2025)	India	Zebrafish—Danio rerio	Mixed‐sex, young (4–6 months), and old (18–20 months)	High‐fat diet (HFD) induced type 2 diabetes model	*n* = 12/group; 5 groups per young and old age cohort (Control, TZP only, diabetic + TZP, diabetic, diabetic + donepezil)	1.2 mg/kg, subcutaneous injection, once daily for 4 weeks (starting at the 10th week of HFD until the 14th week)	After 6 weeks HFD (hyperglycemia established)	8 weeks total (6 weeks HFD + 2 weeks treatment)	Post‐treatment Day 14 (behavior and molecular)	T‐maze, Novel tank diving, Inhibitory avoidance, improved learning/memory/anxiety metrics	Brain histology examined; not detailed in abstract	Increased GSH, CAT; Increased IL‐10; improved lipid profile; decreased oxidative stress (MDA); measured IL‐1β, TNF‐α, Bcl‐2/Bax ratio (apoptosis marker), GSK‐3β, AMPK	Increased AMPK; potential PI3K/Akt/GSK β‐involvement	Lower glucose; lower adverse metabolic parameters; no mortality reported	TZP ameliorated HFD related cognitive impairment in zebrafish with antioxidant, anti‐inflammatory and anti‐apoptotic effects	Non‐mammalian model; amyloid/tau pathology not assessed
Ma et al. (2024)	China	C57BL/6J Mice	Male, aged 8 weeks, weight 20–25 g	HFD related cognitive impairment	Total *n *= 144 across parts; typical *n *= 6/group for assays; NC group (normal diet) (36), a high‐fat diet group (36), (HFD + TZP) group (*n* = 36), stereotactic injection with lentivirus scramble control shRNA (shC) group (*n* = 6), stereotactic injection with Sh‐SIRT3 lentivirus (sh‐SIRT3) group (*n* = 6), (HFD + TZP + shC) group (*n* = 12), (HFD + TZP + sh‐SIRT3) group (*n* = 12)	1.2 mg/kg, dissolved in normal saline) once a day began at the 10th week for 4 weeks	Weeks 10–14 of HFD (after impairment established)	14 weeks total; 4 weeks treatment	End of week 14 (behavior, molecular)	Morris water maze, improved learning (escape latency), and probe performance (platform crossings)	Nissl staining; synaptic protein expression (MAP‐2, Synaptophysin, PSD95)	Decreased IBA1, GFAP; IL‐1β, IL‐6, TNF‐α, and NLRP3; increased SIRT3; oxidative stress markers improved	SIRT3‐NLRP3 axis (sh‐SIRT3 abrogated benefits)	Reduce body weight; lower blood glucose	TZP attenuates HFD induced cognitive impairment by reducing oxidative stress and neuroinflammation via SIRT3‐NLRP3 signaling	HFD model; daily dosing; generalizability to human AD uncertain
Yang 2024—in vivo (Yang et al. 2024)	China	APP/PS1 double transgenic mice	6 months old, mixed sex	Alzheimer's disease model (APP/PS1)	*n* = 10/group (WT vs. APP/PS1 ± TZP)	10 nmol/kg intraperitoneal weekly for 8 weeks	After pathology established (6 months)	8 weeks treatment	End of treatment (behavior, histology, molecular assays)	Morris water maze improved learning and memory	Decreased Amyloid plaques (Thioflavin‐S), decreased GFAP astrocytosis	Decreased BACE1; Increased GLUT1, HK, G6PD, PFK, ATF4, SCAF1, GluN2A/B; decrease apoptosis markers	GLP‐1R mediated, improved glucose metabolism, mitochondrial and synaptic support	Reduced weight gain; no increased mortality	TZP improved cognition, reduced plaques, restored glucose metabolism and synaptic proteins in APP/PS1 mice	Single dose level, only APP/PS1 model, no tauopathy assessment
Hassan et al. (2024)	Egypt	Wistar Albino rats	Male, weight 200–250 g	Colistin‐induced nephro and neurotoxicity	*n* = 12/group; 3 groups (control; colistin; colistin + TZP)	1.35 mg/kg subcutaneous on Days 1, 4, 7 (with colistin 300,000 IU/kg/day intraperitoneal—7 days)	Coadministered, 1 h after daily colistin	7 days (+24 h assessments)	24 h post last dose (behavior, labs, and histology)	Open field ambulation, immobility	Renal H&E/PAS injury scores; brain GFAP and Bax IHC; Purkinje cell counts; improved morphology	Decreased KIM‐1, NGAL, creatinine; decreased NF‐κB p65, TNF‐α; increased Nrf2, GSH; decreased MDA; decreased ER stress (ATF4, CHOP); increased p‐CREB/BDNF/TrkB; modulated PI3K/p‐Akt/GSK3β	PI3K/p‐Akt/GSK3β, NF‐κB, Nrf2, p‐CREB/BDNF/TrkB; ER stress attenuation	Attenuated colistin nephrotoxicity (lower creatinine); no mortality reported	As adjuvant to colistin, TZP mitigated renal and neuronal toxicity with anti‐inflammatory, antioxidant, anti‐apoptotic effects	Short duration; antibiotic toxicity model rather than neurodegeneration
Forny Germano et al. (2024)	Canada	5XFAD, APP/PS1 transgenic and WT littermates Mice	Males and females 6–12 months of age	Transgenic Alzheimer's models (amyloid pathology)	Not specified	Subcutaneous once daily; 2.5–5 to 10 nmol/kg for weeks 1, 2, and 3 till 7, respectively (6‐month cohort)	After pathology established	6 weeks for young and 7 weeks for adults	End of treatment (behavior, histology, transcript)	Open field, Novel object recognition, Morris water maze	Thioflavin amyloid plaque counts; IBA1/GFAP immunostaining no change	Hippocampal inflammatory/degeneration mRNAs showed no reduction	Metabolic engagement confirmed (reduce weight, improved GTT); no CNS biomarker modulation detected	Reduce body weight; improved glucose tolerance	No detectable improvement in structural/functional neurodegeneration parameters despite metabolic effects	Daily dosing; treatment window; amyloidâ€‘only models; may not capture tau or vascular pathology
Guo et al. (2023)	China	Sprague‐Dawley rats	Male, aged 7–8 weeks, weighing between 180 and 200 g	HFD and streptozotocin injection induced diabetes with cognitive impairment	Flowchart: Con (*n* = 12), Con + TZP (*n* = 12), DM (*n* = 16), DM + TZP (*n* = 15)	1.35 mg/kg once weekly, intraperitoneal for 10 weeks	After diabetes induction; weekly through to Week 15	15 weeks protocol (OGTT Week 13; MWM week 14; sacrifice Week 15)	Weeks 13–15	Morris water maze, improved escape latency, and platform crossings	Nissl staining; Golgi staining, dendritic spine density	Decrease Aβ accumulation, Synaptic proteins (PSD95, SYT1), TNF‐α, IL‐6, IL‐1β, restored PI3K/Akt/GSK3β signaling	PI3K/Akt/GSK3β; insulin signaling normalization	Lower fasting glucose; improved insulin; body weight reduced	Weekly TZP ameliorated diabetic cognitive deficits via anti‐inflammatory, synaptoprotective and insulin‐signaling pathways	Single weekly dose; translational dosing differs from clinic; rat diabetes model

Abbreviations: 5XFAD, five familial Alzheimer's disease mutations (mouse model); ACSL4, Acyl‐CoA synthetase long chain family member 4; AD, Alzheimer's disease; Akt, protein kinase B; AMPK, AMP‐activated protein kinase; APP/PS1, amyloid precursor protein/presenilin 1 (Alzheimer's model); ATF4, activating transcription factor 4; ATP, adenosine triphosphate; Aβ, amyloid beta; BACE1, beta‐site APP‐cleaving enzyme 1; BALB/c, Bagg albino (mouse strain); Bax, Bcl‐2‐associated X protein; Bcl‐2, B‐cell lymphoma 2; BDNF, Brain‐Derived neurotrophic factor; C57BL/6, C57 black 6 (mouse strain); CAT, catalase; CHOP, C/EBP homologous protein; CNS, central nervous system; DA, dopamine; DM, diabetes mellitus; Drp1, dynamin‐related protein 1; ELISA, enzyme‐linked immunosorbent assay; Fer‐1, ferrostatin‐1 (ferroptosis inhibitor); G6PD, glucose‐6‐phosphate dehydrogenase; GFAP, glial fibrillary acidic protein; GLP‐1R, glucagon‐like peptide‐1 receptor; GluN2A/B, glutamate ionotropic receptor nmda type subunit 2A/2B; GPX4, glutathione peroxidase 4; GSH, glutathione; GSK‐3β, glycogen synthase kinase 3 beta; GSSG, glutathione disulfide; GTT, glucose tolerance test; HFD, high‐fat diet; HK, hexokinase; HPLC, high performance liquid chromatography; i.p., intraperitoneal; IHC, immunohistochemistry; IL‐10, interleukin‐10; IL‐1β, interleukin‐1 beta; KIM‐1, kidney injury molecule‐1; MDA, malondialdehyde; MPTP, 1‐methyl‐4‐phenyl‐1,2,3,6‐tetrahydropyridine (neurotoxin); MWM, Morris water maze; NC, normal chow (diet); NF‐κB, nuclear factor kappa‐light‐chain‐enhancer of activated B cells; NGAL, neutrophil gelatinase‐associated lipocalin; NLRP3, NLR family pyrin domain containing 3; Nrf2, nuclear factor erythroid 2–related factor 2; OE, overexpression; OGTT, oral glucose tolerance test; p62, sequestosome 1; Parkin, Parkin RBR E3 ubiquitin protein ligase; PAS, periodic acid‐schiff (histological stain); p‐CREB, phosphorylated cAMP‐response element‐binding protein; PD, Parkinson's disease; PFK, phosphofructokinase; PI3K, phosphoinositide 3‐kinase; Pink1, PTEN‐induced putative kinase 1; PSD95, postsynaptic density protein 95; pSer129 α syn, phosphorylated serine 129 alpha‐synuclein; ROS, reactive oxygen species; SCAF1, supercomplex assembly factor 1; shC, scramble control shRNA; shRNA, short hairpin RNA; sh‐SIRT3, sirtuin 3 knockdown (via shRNA); SIRT3, sirtuin 3; SLC7A11, solute carrier family 7 member 11; SOD, superoxide dismutase; SOX2, SRY‐box transcription factor 2; SYT1, synaptotagmin 1; TH, tyrosine hydroxylase; TNF‐α, tumor necrosis factor alpha; TrkB, tropomyosin receptor kinase B.; TZP, tirzepatide; WB, western blot; WT, wild type; α‐Syn, alpha‐synuclein.

Regarding MPTP‐induced Parkinsonism (male C57BL/6 mice) (Tian et al. 2025), 4 weeks of TZP after toxin exposure improved motor performance and preserved dopaminergic neuronal integrity, with molecular readouts consistent with improved mitochondrial quality control, including modulation of Drp1 and increased Pink1/Parkin/p62 signaling. In rotenone Parkinsonism (Wistar rats) 28, daily subcutaneous TZP (50 and 100 nmol/kg for 28 days) improved locomotor activity and motor coordination, increased striatal dopamine, and reduced inflammatory cytokines and oxidative stress markers, with the strongest effects at 100 nmol/kg.

In metabolic–cognitive impairment, TZP improved behavioral performance and inflammatory/oxidative profiles. In an HFD zebrafish Type 2 diabetes model (Misra et al. 2025), once‐daily subcutaneous TZP administered after hyperglycemia was associated with improved learning/memory and anxiety‐related measures alongside antioxidant and anti‐inflammatory changes, with AMPK‐related signaling reported. In an HFD mouse cognitive‐impairment model (C57BL/6J) (Ma et al. 2025), daily TZP (1.2 mg/kg) improved Morris water maze outcomes, increased synaptic proteins, reduced microglial and astrocytic activation and inflammatory mediators (including NOD‐like receptor family pyrin domain containing 3 [NLRP3]), and increased sirtuin 3 (SIRT3).

As for Alzheimer‐type models, findings were mixed. In APP/PS1 mice (Yang et al. 2024), weekly TZP (10 nmol/kg intraperitoneal for 8 weeks) improved Morris water maze performance and was accompanied by reductions in plaque‐related and astrocytic markers and restoration of metabolic and synaptic readouts. By contrast, in 5xFAD and APP/PS1 cohorts (Forny Germano et al. 2024), escalating daily TZP dosing produced metabolic engagement (weight loss and improved glucose tolerance) without detectable improvements in behavioral outcomes or amyloid/inflammatory neuropathology measures, representing the principal null CNS benefit signal among the included animal studies.

In injury models with brain involvement, TZP also showed protective signals. In colistin‐induced nephro‐ and neurotoxicity (Wistar rats) (Hassan et al. 2024), an adjuvant TZP regimen reduced inflammatory, oxidative, and ER‐stress markers and increased p‐CREB/BDNF/TrkB signaling, with pathway readouts implicating PI3K/p‐Akt/GSK‐3β and NF‐κB/Nrf2‐related biology. In diabetes‐related cognitive deficit (Sprague–Dawley rats) (Guo et al. 2023), weekly TZP (1.35 mg/kg intraperitoneal for 10 weeks) improved Morris water maze performance, reduced inflammatory cytokines and amyloid beta (Aβ)‐related measures, improved synaptic markers and dendritic spine indices, and restored PI3K/Akt/GSK‐3β signaling (Table 2).

### 3.4 Clinical Studies

#### 3.4.1 Cohort Studies

Three retrospective propensity score‐matched cohort studies using the TriNetX database were included (Table 3). Lin et al. (2025) evaluated adults with Type 2 diabetes (30,340 GLP‐1 RA group, including TZP users matched to 30,340 controls not receiving GLP‐1/GIP agonists) with outcomes assessed at 1, 3, 5, and 7 years. TZP exposure was associated with lower incidence of neurodegenerative outcomes and ischemic stroke compared with matched controls, whereas the dementia‐risk reduction was reported as nonsignificant; all‐cause mortality was lower, and intracerebral hemorrhage showed a nonsignificant trend. Although the primary matched comparison pooled GLP‐1RAs users, Lin et al. (2025) additionally reported a direct head‐to‐head comparison of TZP versus semaglutide in their Supporting Information, from which TZP‐specific estimates were extracted. Azzam et al. (2025) included adults with IIH (193 TZP‐exposed matched to 193 controls) with follow‐up at 3, 6, 12, and 24 months. At 24 months, TZP use was associated with lower risks of papilledema, visual disturbance/blindness, and headache, alongside a significant reduction in BMI. Toraih et al. (2025) evaluated vestibular outcomes in a large matched cohort (77,259 TZP users matched to 77,259 controls not receiving GLP‐1RAs), with outcomes assessed at 6 months, 1 year, and 3 years; comparative analyses versus semaglutide users were also reported. TZP showed lower absolute risk than semaglutide but relatively higher rates of central vertigo and vestibular neuronitis.

**TABLE 3:** Summary of the included cohort studies.

Study ID	Country	Study design	Sample size (exposure vs. comparator)	Data collection	Follow‐up timepoints	Objectives	Inclusion criteria	Exclusion criteria	BMI (no. (%)/mean ± SD)	Age (mean)	Male no. (%)	Comparator (placebo, other specific GLP‐1 agonist, etc)	Outcomes	Efficacy findings	Safety findings	Limitations
Lin et al. (2025)	TriNetX dataset across the United States, Europe, Asia‐Pacific, and Latin America	Retrospective propensity score‐matched cohort study (TriNetX)	GLP‐1RA 30,340 vs. matched controls 30,340 (Other antidiabetics)	December 2017–December 2024 data (7 years)	1, 3, 5, 7 years	To evaluate whether GLP‐1RA, including TZP, reduces the risk of neurodegenerative disorders and stroke compared to controls	Adults ≥ 40 years with T2DM and obesity who received antidiabetic drugs, including GLP‐1RA, with no prior diagnosis of neurodegenerative disease or stroke before baseline and had at least 1 year of follow‐up after the index prescription	History of T1DM, neurodegenerative disease, and prior stroke before the index date, prior use of other GLP‐1RAs then switched to TZP or semaglutide within 3 months of initiating another antidiabetic treatment	(30–40 kg/m^2^); GLP‐1RA group: 409 (2.5%), controls: 437 (2.9%)/(>40 kg/m^2^); GLP‐1RA group: 280 (2.4%), controls: 286 (2.4%)	GLP‐1RA: 57.9 ± 9.9; control: 58 ± 10.8	Out of 30,430 after matching, TZP: 13,017 (42.8) vs. control: 13,083 (43)	Matched patients with T2DM receiving standard care but not GLP‐1/GIP agonists	Incidence of neurodegenerative disorders (Alzheimer's, Parkinson's, dementia) and ischemic stroke	During a 7‐year follow‐up, TZP significantly reduced the risk of neurodegenerative diseases, ischemic stroke, and all‐cause mortality. In addition, TZP showed benefits towards a lower risk of dementia, but with no significance	TZP showed a significant reduction in all‐cause mortality and a nonsignificant trend towards intracerebral hemorrhage	observational study, residual confounding (e.g., frailty, functional status) cannot be excluded, risking healthy‐user or selection bias. TriNetX lacks genetic and neuroimaging data, limiting mechanistic insights. Medication exposure was inferred from prescriptions without confirmation
Azzam et al. ()	TriNetX dataset across the United States, Europe, Middle East: “Australia, Belgium, Brazil, Bulgaria, Estonia, France, Georgia, Germany, Ghana, Israel, Italy, Japan, Lithuania, Malaysia, Poland, Singapore, Spain, Taiwan, United Arab Emirates, and the United Kingdom”	Retrospective propensity score‐matched cohort study (TriNetX)	193 TZP‐exposed vs. 193 matched controls (standard care)	From Inception till Nov 2024 (2 years)	3, 6, 12, 24 months	To assess efficacy of TZP in idiopathic intracranial hypertension (IIH) patients	Adults ≥18 years with IIH diagnosis, standard therapy participation, and complete baseline data, including BMI measurements	Secondary intracranial hypertension, including cerebral venous thrombosis and structural neurological abnormalities, prior incretin therapy within 12 months, pregnancy/post‐partum, insufficient follow‐up (<6 months)	TZP: 41.3 ± 8.82; controls: 38.7 ± 9.33	TZP: 37.7 ± 8.52; control: 37.5 ± 8.78	Out of 193 after matching, TZP: 10 (5.18) vs. control: 10 (5.18)	Standard IIH care (acetazolamide, topiramate, and weight management), no GLP‐1/GIP therapy	Papilledema severity, visual function, headache frequency, treatment resistance, BMI changes	At 24 months: 68% lower papilledema risk, 73.9% lower visual disturbance/blindness risk, 19.7% lower headache risk; significant BMI reduction (−1.15 kg/m^2^)	Progressive BMI reduction in the TZP group, starting with a modest decrease at 3 months, achieving maximal effect at 24 months with −1.15 kg/m^2^	TriNetX structure limited dosing and adherence analyses. Retrospective design risks unmeasured confounders, whereas nonstandard ICP measures, variable follow‐up, and incomplete co‐medication data may affect generalizability
Toraih et al. (2025)	TriNetX dataset	Retrospective propensity score‐matched cohort study (TriNetX)	TZP users 77,259 vs. matched controls 77,259 (Not received GLP‐1RAs)	January 2018–October 2024 (7 years)	6 months, 1 year, 3 years	To investigate the association between GLP‐1RA, including TZP use, and the risk of vestibular disorders	Adults ≥18 years with diabetes or obesity and newly prescribed semaglutide or TZP	NR	TZP: 31,395 (40.6%); controls: 31,375 (40.6%)	TZP: 51.9 ± 13.7; controls: 51.9 ± 13.7	TZP: 25,991 (33.6%) controls: 26,900 (34.8%)	1:1 propensity‐score‐matched controls with similar indications who did not receive GLP‐1RAs. TZP vs. Semaglutide users	New‐onset vestibular disorders (ICD‐10 H81 and subcodes) at 6 months, 1 year, 3 years; time‐to‐event via Cox models; Subtype distribution (e.g., BPPV, Menieres' disease, vestibular neuronitis, central origin vertigo) and time‐to‐diagnosis	NA	TZP showed lower absolute risk than semaglutide, but relatively higher rates of central vertigo and vestibular neuronitis. TZP users had a cumulative incidence of vestibular disorders of 0.10% at 6 months, 0.15% at 1 year, and 0.19% at 3 years (vs. 0.04%–0.15% in controls). Hazard ratios were 3.19, 4.26, and 4.55 at 6 months, 1 year, and 3 years, respectively. The most common subtype was BPPV (58.4%), followed by peripheral vertigo (14.6%), Ménière's disease (12.2%), central‐origin vertigo (8.6%), and vestibular neuronitis (5.8%). Median onset was 3.4 months for BPPV, 4.6 months for neuronitis, and 3.8 months for central vertigo	No data on dosing regimens or treatment duration; could not assess dose response; reliance on ICD‐10 coding; potential underreporting/misclassification; residual confounding cannot be excluded despite propensity matching; potential ascertainment bias

#### 3.4.2 Case Reports

Eight reports (Wentling et al. 2025; Abdi et al. 2025; Bodanowitz et al. 2025; Parikh et al. 2024; Bobbs and Howland 2024; Jamail et al. 2024; Tucker and Ritchie 2024) described nine individual cases after TZP exposure. Most patients were female and were treated for obesity or overweight. Presentations clustered as three common peroneal neuropathy with foot drop, one autoimmune encephalitis with anti‐NMDA receptor antibodies, one status epilepticus from severe hypoglycemia, one valproic acid toxicity with encephalopathy, one psychiatric reaction with paranoia and visual hallucinations after a compounded product, and one migraine with hypoglycemia and possible mitochondrial dysfunction. Onset ranged from within 24 h to about 11 months after initiation. We summarize the neurologic presentations here and refer other events to Table 4.

**TABLE 4:** Summary of case reports reported neurological conditions to tirzepatide.

Study ID	Country	Patient demographics	Primary disease	Clinical presentation and diagnosis	Timing after TZP given	Dosage when ADR occurs	First time intervention measures	Investigations	Management	Improvement after managing	Return time	Drug rechallenge	Combined medication	Brief summary
Wentling et al. (2025)	USA	58‐year‐old female	Obesity (weight loss goal)	Left foot drop, neuropathic pain; common peroneal neuropathy	After ∼4 months on TZP	Not reported; treated for 6 months, weight loss 34.5 lbs	Physical therapy first (unsuccessful); gabapentin added	EMG/NCS: conduction block at fibular head; MRI lumbar showed mild degenerative changes only	Gabapentin 100 mg TID, PT, spring‐leaf AFO	Yes, gait improved	Not reported	Continued TZP (not withdrawn)	None reported	Rapid TZP‐associated weight loss led to compressive neuropathy (slimmer's paralysis)
Abdi et al. (2025)	UAE	18‐year‐old female	Obesity/overweight (BMI 27.7 → 25.2)	Confusion, agitation, psychiatric symptoms, speech impairment, seizures; anti‐NMDA autoimmune encephalitis	About 2 weeks after last (fifth) injection	2.5 mg weekly for 5 weeks	IV levetiracetam 2 g given acutely	MRI: right amygdala enlargement/hyperintensity; CSF positive for anti‐NMDA antibody, oligoclonal bands; EEG normal; pelvic US/MRI negative for teratoma	IV methylprednisolone 5 g over 5 days, IVIG 140 g over 7 days, anti‐seizure meds (levetiracetam, phenytoin, lacosamide), monthly IVIG × 6 months	Yes, seizure‐free after therapy	Not precisely stated; improved at discharge, seizure‐free follow‐up	Not reported	None	First case of TZP‐associated anti‐NMDA autoimmune encephalitis (Naranjo score 5, probable)
Bodanowitz et al. (2025)	Germany	66‐year‐old female	Weight loss (off‐label use, BMI 25.3)	Weakness, nausea, vomiting, rhabdomyolysis (CK 4249 U/L, myoglobin 472 ng/mL)	Symptoms started within 24 h of first dose	15 mg (supratherapeutic starting dose; recommended 2.5 mg)	Stopped TZP, IV fluids	CK, myoglobin, LFTs elevated; renal function normal	IV crystalloids, antiemetics, monitoring	Yes, full resolution in 3–4 days	No recurrence at 8‐week follow‐up	No rechallenge	On atorvastatin, gabapentin, amitriptyline (stable >3 years)	Second reported rhabdomyolysis with TZP; linked to supratherapeutic dosing plus statin use
Parikh et al. (2024)	USA	27‐year‐old female	Weight loss (no T2DM), prior seizure history	Severe hypoglycemia (glucose 52–55 mg/dL) with status epilepticus	Within 2 weeks of starting TZP	15 mg weekly, from unofficial source (improper escalation)	IV dextrose, midazolam, IV levetiracetam, airway protection	Labs: glucose 52 mg/dL, imaging normal, EEG normal, MRI brain normal	ICU care, sedation, later dose reduced to 5 mg weekly	Yes, recovered fully; seizure‐free at 6 months	Follow‐up 6 months seizure‐free	Dose reduced, not stopped (continued at 5 mg weekly)	None reported	Case highlights severe hypoglycemia and seizure risk from unregulated maximum dosing
Bobbs and Howland (2024)	USA	61‐year‐old female	Overweight with no comorbidities	Headache, vomiting, diarrhea, paranoia, visual hallucinations	After dose increase of compounded TZP	Not specified (compounded formulation, increased dose)	Advised to discontinue TZP	Labs showed transaminitis	Supportive, discontinuation of drug	Yes, hallucinations/paranoia resolved	8 days later patient symptom‐free	No rechallenge	History of anxiety and depression	First reported case of paranoia and hallucinations after compounded TZP; off‐label use for weight loss
Jamail et al. (2024)	USA	43‐year‐old female	Obesity, BMI 32, no diabetes	Migraines and hypoglycemia, possible mitochondrial dysfunction	Hypoglycemia, persistent migraines with aura	Not specified (after exposure to semaglutide and tirzepatide injections)	Not reported	Diet modification and supplements (CoQ10, D‐ribose, B‐complex, acetyl‐l‐carnitine)	No detailed labs reported; clinical observation of hypoglycemia	Nutritional and supplemental therapy	Yes, blood sugars stabilized, migraines resolved	Not reported	Not attempted	Migraines and hypoglycemia after TZP
Tucker 2024—case 1 (Tucker and Ritchie 2024)	USA	77‐year‐old male	Obesity, prediabetes, CAD, HLD, CKD 3a, CHF, prior lumbar surgery, knee arthroplasty	Bilateral foot drop; bilateral common peroneal neuropathy	Within 6–8 months of starting TZP	Not reported; started standard titration Feb 2023, reached weight loss 24% by 6–8 months	Orthotics, physical therapy initiated	Labs unremarkable; MRI lumbar showed stenoses but not causal; EMG showed bilateral peroneal neuropathy; nerve ultrasound with mild increase vascularity	Conservative: orthotics, PT; considering surgical release	Not reported	Not reported	No	On cardiac and CKD medications (not detailed in case)	Bilateral peroneal neuropathy (slimmer's paralysis) after rapid 24% weight loss
Tucker 2024—case 2 (Tucker and Ritchie 2024)	USA	56‐year‐old female	Type 2 diabetes, depression, anxiety, SLE, Sjögren's, ADHD	Right then left foot drop; bilateral peroneal neuropathies	Right: ∼7 months after switch to TZP (Apr 2023); Left: ∼11 months after (August 2023)	Escalated from 2.5 to 15 mg weekly (over months)	Orthotic + right peroneal release in May 2023	Labs: A1C 4.7%; copper mildly elevated, vitamin B6 low; MRI lumbar normal; EMG confirmed peroneal neuropathies	Right peroneal release (May 2023), left release (September 2023), AFO, PT	Yes, clinical improvement after decompression	By Sep 2023 showed improvement	Continued TZP (not withdrawn)	Past semaglutide use (2019–2022); multiple comorbid meds	Bilateral peroneal neuropathy after 28% body weight loss; improved with decompression
Cruz Rivera et al. (2023)	USA	50‐year‐old male	Epilepsy, on divalproex ER 1500 mg BID + levetiracetam 1000 mg daily	Malaise, anorexia, ataxia, encephalopathy, petechial rash; valproic acid toxicity	After 3 weekly doses	2.5 mg once weekly	Held valproate, started levocarnitine	VPA 220 mcg/mL, ammonia 63, platelets 40, Na 126	Supportive care, levocarnitine, VPA withheld	Yes, discharged well by Day 6	6 days hospitalization	Not reported	Divalproex ER, levetiracetam	Likely interaction: TZP slowed gastric emptying and displaced protein binding, raising free VPA levels

Abbreviations: A1C, glycated Hemoglobin; ADHD, attention deficit hyperactivity disorder; AFO, ankle‐foot orthosis; BID, twice a day; BMI, Body mass index; CAD, coronary artery disease; CHF, congestive heart failure; CKD, chronic kidney disease; CSF, cerebrospinal fluid; EEG, electroencephalogram; EMG/NCS, electromyography/nerve conduction studies; ER, extended release; HLD, hyperlipidemia; IV, intravenous; IVIG, intravenous immunoglobulin; MRI, magnetic resonance imaging; Na, sodium; NMDA, N‐methyl‐D‐aspartate; PT, physical therapy; SLE, systemic lupus erythematosus; TID, three times a day; TZP, tirzepatide; UAE, United Arab Emirates; US, ultrasound; VPA, valproic acid.

### 3.5 Synthesis of Results

Overall, the mapped evidence indicates that TZP is most consistently associated in preclinical models with improved mitochondrial and bioenergetic indices and reduced oxidative and inflammatory injury, with recurrent involvement of mitophagy and PI3K/Akt‐related signaling. In vivo findings were broadly directionally concordant across Parkinsonian toxin models, metabolic and diabetes‐related cognitive impairment, and injury models, although one Alzheimer‐type transgenic study reported metabolic effects without detectable CNS benefit. Clinical cohort studies provided observational associations compatible with these signals and suggested a low absolute frequency of vestibular diagnoses, while remaining subject to residual confounding. Case reports did not quantify risk but clustered in recognizable contexts, particularly compressive peroneal neuropathy after marked weight loss, autoimmune encephalitis, and severe hypoglycemia in nonstandard or unregulated dosing settings.

### 3.6 Critical Appraisal

Cohort studies received high NOS ratings, with all three scoring 8/8 stars (Table S1). Across eight case reports describing nine cases, seven cases scored 8/8 on the JBI checklist, whereas Bobbs and Howland (2024) and Jamail et al. (2024) each scored 7/8 (Supporting Information File 1). All five in vitro studies were rated “reliable with restrictions” on ToxRTool, with total scores ranging from 15 to 17 (Table S2). For in vivo studies, six studies were of high quality, scoring nine or more, whereas three studies showed low quality, scoring less than five on CAMARADE checklist. Though on SYRCLE checklist, judgments were dominated by “unclear” ratings across several domains, indicating some limitations in reporting and internal validity (Supporting Information File 1).

## 4 Discussion

### 4.1 Overview of Main Findings

In this scoping review, we mapped current preclinical and clinical evidence related to the neurological profile of TZP. Twenty‐two studies met the inclusion criteria as follows: 3 retrospective human cohort studies, 11 preclinical investigations in animal and cell models, and 9 clinical case reports. Preclinical studies in models of PD, AD, stroke, and metabolic‐cognitive dysfunction consistently showed mitigation of neuropathological features as well as improvement of disease phenotypes. However, no clinical trials or observational studies have established clinical efficacy for PD or AD in humans. Clinical data remained limited to metabolic and broad neurological endpoints. Case reports described various neurological or neurometabolic events, most resolved after intervention or dose adjustment. The evidence suggests a biologically credible but clinically underexplored neuroprotective profile for TZP, meriting further mechanistic and translational research. The current evidence can inform future hypothesis or exploratory clinical trials rather than justify efficacy assumptions at this stage. Throughout this synthesis we distinguish direct neuroprotection, which requires central nervous system penetration and engagement of neuronal or glial receptors, from indirect neurological benefit mediated by peripheral metabolic and vascular effects such as improved glycemia, weight loss, blood‐pressure lowering, and endothelial function. This distinction is clinically important, because several of the human signals discussed below are plausibly explained by systemic mechanisms without invoking direct central action.

### 4.2 Cellular Studies and Molecular Plausibility (In Vitro)

In neuronal/astrocytic systems exposed to Aβ, TZP conferred GIP/GLP‐1R‐dependent protection, preserving mitochondrial membrane potential, restoring ATP, and stabilizing mitochondrial markers, consistent with a bioenergetic rescue at the cell level (Yang et al. 2024). In human brain microvascular endothelium subjected to OGD/R, TZP increased TEER, reduced permeability, and restored tight‐junction proteins via C/EBP‐α‐mediated Claudin‐1 upregulation, pinpointing a BBB‐stabilizing mechanism (Wang, Wang, et al. 2025). Under high‐glucose conditions modeling neuronal insulin resistance, TZP activated pAkt/CREB/BDNF, normalized GLUT3/GLUT4 and related transcripts, and reversed adverse epigenetic/miRNA shifts in neurotrophic programs—indicating restoration of metabolic trophic coupling in neurons (Fontanella et al. 2024).

These convergent cellular mechanisms, mitochondrial protection, BBB reinforcement, and neurotrophic/insulin‐signaling support are congruent with the broader GIP/GLP‐1RA neurobiology documenting reduced neuroinflammation, improved synaptic function, and engagement of CREB/BDNF pathways across neurodegenerative contexts (Athauda and Foltynie 2016; Nowell et al. 2023). They also dovetail with pharmacologic insights showing TZP's dual GIP/GLP‐1 receptor engagement. They provide a plausible basis for additive or complementary actions on neuronal and endothelial targets (Zhao et al. 2022; Liu 2024).

These wide molecular effects, spanning mitochondrial, inflammatory, and neurotrophic pathways, should be interpreted as hypothesis‐generating. Therefore, clinical trials investigating dual GIP/GLP‐1 RAs are warranted to provide clear evidence of their efficacy.

### 4.3 Mechanistic Coherence in Preclinical Evidence (In Vivo)

Across toxin, ischemic, and metabolic‐cognitive animal models, TZP engaged neuroprotective pathways relevant to neuronal survival and circuit function. In an MPTP Parkinson's model, TZP preserved dopaminergic neurons and improved motor behavior while restoring mitochondrial energy balance (Increased ATP), normalizing mitophagy‐associated proteins (Increased Pink1, Parkin, and p62), and reducing Drp1—together indicating rescue of mitochondrial quality control and excessive fission (Tian et al. 2025). These effects align with the broader GLP‐1RA literature in PD, where agents such as liraglutide restore PGC‐1α–linked biogenesis, dynamics (fusion/fission), and autophagy with dopaminergic protection, supporting a mechanistic bridge between incretin signaling and mitochondrial/autophagic homeostasis in nigrostriatal neurons (Wu et al. 2022).

In ischemic stroke models, post‐insult TZP reduced infarct volume, improved neurological scores, and stabilized the BBB by upregulating Claudin‐1 through C/EBP‐α activation; disruption of this axis abrogated protection in endothelial assays, strengthening causal inference at the neurovascular interface (Wang, Wang, et al. 2025). The brain‐partitioning values reported in that model (*K*_p, brain_ ≈ 0.12–0.14; Table 2) should nonetheless be interpreted with caution as they derive from a middle cerebral artery occlusion paradigm in which the BBB is already disrupted and cannot be extrapolated to the intact human central nervous system. TZP is a large (∼4.8 kDa) acylated peptide with limited expected passive penetration, and its central effects may be substantially mediated by indirect routes such as through vagal afferent signaling and action at circumventricular organs rather than by direct parenchymal entry (Nowell et al. 2023). In metabolic‐cognitive paradigms, TZP improved learning and memory while attenuating oxidative stress and neuroinflammation, notably via SIRT3–NLRP3 signaling in high‐fat diet mice and PI3K/Akt/GSK‐3β restoration in diabetic rats, alongside synaptic support (e.g., ↑PSD‐95/synaptophysin, ↑spine density). Behavioral gains tracked with glycemic and weight improvements, consistent with energy–synapse coupling (Ma et al. 2025; Guo et al. 2023).

Importantly, an incretin‐biology laboratory found that neither TZP nor semaglutide altered amyloid pathology, neuroinflammation, behavior, or cognition despite clear peripheral metabolic engagement in 5xFAD and APP/PS1 transgenic Alzheimer's models (Forny Germano et al. 2024). This null result warrants substantial weight and should temper extrapolation from the smaller positive studies. Moreover, in amyloid‐transgenic Alzheimer's models, TZP produced peripheral metabolic engagement without apparent central biomarker change, underscoring dose, regimen, timing, and disease‐stage dependencies and motivating standardized in vivo readouts (behavioral batteries; synaptic, glial, and mitochondrial panels; and BBB metrics) for cross‐study comparability (Hristov et al. 2025).

### 4.4 Human Data: Preliminary but Encouraging

Human evidence is presently limited to retrospective, EHR‐based cohorts. However, it broadly supports potential neurological benefits with incretin therapy and provides early, TZP‐specific signals. In a federated TriNetX analysis covering 2017–2024 with propensity‐matched comparators and follow‐up at 1, 3, 5, and 7 years, exposure to GLP‐1RAs—including TZP—was associated with lower incidence of neurodegenerative disorders and ischemic stroke and reduced all‐cause mortality (Lin et al. 2025). The reduced incidence of ischemic stroke in this setting is, however, more likely to be due to TZP established systemic cardiovascular and metabolic effects, blood‐pressure reduction, lipid improvement, and better endothelial function (Sinha et al. 2023; Syed 2022), rather than to direct central neuroprotection, illustrating the direct‐versus‐indirect distinction drawn above. These observations align with other real‐world studies linking GLP‐1RA use to lower dementia (Tang et al. 2024) and Alzheimer's disease (Wang, Wang, et al. 2024) risk at the population level.

TZP‐specific findings emerged in a TriNetX cohort of IIH, where matched patients receiving TZP experienced substantially reduced papilledema and visual disturbance/blindness risk, fewer headaches, and progressive BMI reduction across 3–24 months (Azzam et al. 2025). These outcomes suggest a combination of metabolic and potential neurovascular benefits relevant to raised intracranial pressure. By contrast, another extensive TriNetX analysis reported small absolute risks but higher relative hazards for vestibular diagnoses among TZP users compared with matched controls (and differing subtype distributions vs. semaglutide), with cumulative incidences on the order of 0.10% at 6 months, 0.15% at 1 year, and 0.19% at 3 years (Toraih et al. 2025). Although absolute risks remained low, the pattern highlights rare neurologic events that merit pharmacovigilance.

Among all neurological indications surveyed, IIH currently represents the strongest near‐term case for clinical translation. IIH uniquely engages both mechanistic axes distinguished above. A plausible direct central mechanism exists. The GLP‐1 receptor is expressed in the choroid plexus, where its activation reduces cerebrospinal fluid secretion and lowers intracranial pressure in rodent models (Botfield et al. 2017). In a RCT, the GLP1‐RA exenatide reduced telemetric intracranial pressure in women with active IIH within 24 h, an effect too rapid to be explained by weight loss alone (Mitchell et al. 2023). Moreover, TZP showed another indirect mechanism. IIH pathophysiology is tightly linked to obesity, precisely the domain in which TZP metabolic efficacy is greatest. With a dedicated randomized trial of TZP in IIH now registered (NCT07191873) (Duke University 2025), this is the most concrete translational opportunity for a dual GIP/GLP‐1 agonist in neurology.

**FIGURE 2:**
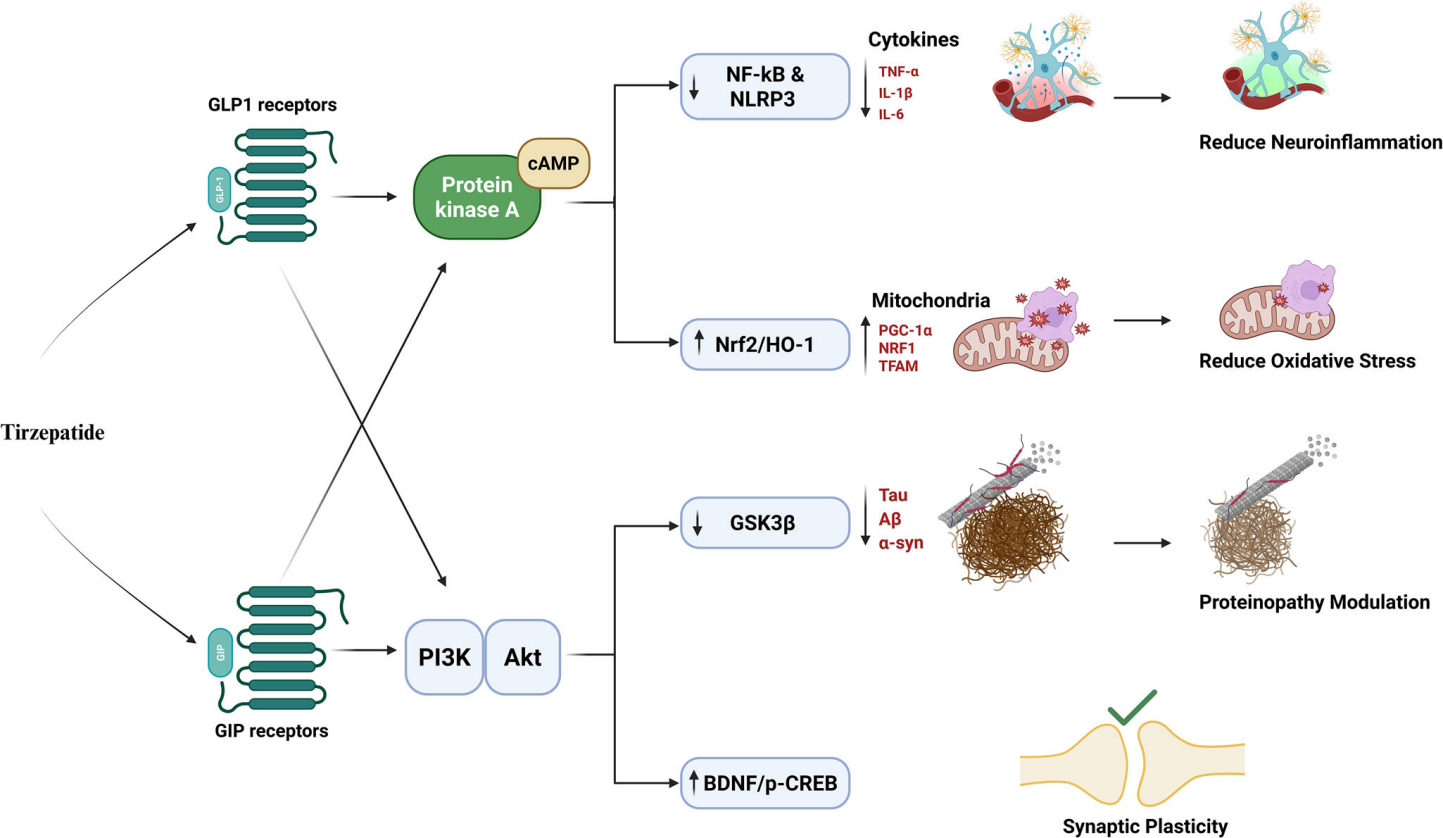
This schematic summarizes the principal neuroprotective mechanisms of tirzepatide identified across the included studies: improved mitochondrial bioenergetics and mitophagy (increased ATP and Pink1/Parkin, and reduced Drp1); reduced oxidative stress and neuroinflammation (SIRT3–NLRP3, NF‐κB); restoration of PI3K/Akt/GSK‐3β and CREB/BDNF signaling; and stabilization of the blood–brain barrier via C/EBP‐α–mediated Claudin‐1 upregulation. BDNF, brain‐derived neurotrophic factor; CREB, cAMP response element‐binding protein; GIP, glucose‐dependent insulinotropic polypeptide; GLP‐1, glucagon‐like peptide 1; NLRP3, NOD‐like receptor family pyrin domain containing; PI3K, phosphatidylinositol‐3 kinase.

Despite extensive preclinical investigations, there are no clinical trials demonstrating that TZP improves clinical outcomes in PD or AD. GIP receptor agonists have a major role in cognition, neurotransmission, and cell proliferation. It shows promising results in synapse protection in models with PD and AD (Zhang and Hölscher 2020) (Figure 2). The current clinical evidence is limited to cohorts of single GLP1‐RA only and link exposure to reduced risks of dementia and PD. However, they do not assess or isolate Dual agonists or provide disease specific outcomes in PD or AD. A recent study found that TZP's dual GIP/GLP‐1 action outperforms GLP‐1 receptor agonist only in lowering mortality and cerebral infarction risks (de Bastos Maximiano et al. 2025).

Some studies that showed negative results should be also considered. In the Phase 3 Exenatide‐PD3 trial (194 patients, 96 weeks), once‐weekly exenatide did not slow motor progression and provided no evidence of disease modification in PD (Vijiaratnam et al. 2025). In the Phase 3 evoke and evoke+ trials (3808 participants with early symptomatic Alzheimer's disease), oral semaglutide did not slow clinical progression on the CDR‐SB over 104 weeks despite improvements in Alzheimer's‐related biomarkers (Cummings et al. 2026). They do not exclude benefit from a mechanistically distinct dual agonist, but they substantially lower the prior probability of success and reinforce that the current TZP evidence is hypothesis‐generating rather than confirmatory.

Therefore, the interpretation should remain cautious. All three cohorts rely on prescription‐inferred exposure (not confirmed ingestion), ICD‐coded outcomes, and variable covariate sets across networks and timepoints. None incorporated neuroimaging, fluid biomarkers, or pre‐specified neurological endpoints (e.g., cognition, quantitative vestibular testing), and important clinical measures—including cardiometabolic panels, hepatic/renal indices, and structured safety outcomes—were generally not enumerated in the extraction fields. Despite propensity matching, residual confounding (e.g., frailty, functional status, socioeconomic and behavioral factors) and ascertainment/miscoding bias cannot be excluded. Accordingly, causal neuroprotection has not yet been established, while the directionality of these observational signals is encouraging and consistent with the known neurobiological plausibility of incretin pathways. Prospective studies with biomarker‐anchored neurological endpoints, standardized exposure definitions, and active‐comparator new‐user designs are needed to clarify clinical impact and better delineate benefit–risk for the nervous system.

### 4.5 Insights From Case Reports

Nine case reports contribute a safety perspective rather than efficacy evidence. The most frequent presentation was common peroneal neuropathy (“slimmer's paralysis”) with new‐onset foot drop after rapid, large‐magnitude weight loss, consistent with mechanical compression at the fibular head rather than direct neurotoxicity; management ranged from orthoses/physical therapy to surgical decompression, with improvement reported (Wentling et al. 2025; Tucker and Ritchie 2024).

Other isolated events spanned distinct mechanisms: (i) autoimmune encephalitis (anti‐NMDA receptor) after standard weekly initiation, treated with IV methylprednisolone and IVIG, with seizure freedom on follow‐up (Abdi et al. 2025), (ii) status epilepticus from severe hypoglycemia after improper first‐line 15 mg weekly dosing from an unregulated source, resolving with dextrose/benzodiazepines and antiseizure therapy; therapy later continued at 5 mg (Aarohi Parikh 2024); and (iii) acute psychiatric reaction (paranoia/visual hallucinations) after compounded TZP with dose increase, resolving after discontinuation (Bobbs and Howland 2024). Notably, onset ranged from within 24 h (rhabdomyolysis) to approximately 11 months (progressive peroneal neuropathies). Dosing errors or rapid escalation (notably 15 mg starts) and polypharmacy (e.g., statins, valproate) were prominent contributors in several cases; rechallenge was uncommon, and continuation at a lower dose versus discontinuation depended on phenotype and response. Reports involving compounded TZP align with current regulatory warnings about unapproved compounded GLP‐1 products and underscore the need to use approved sources only (FDA 2025).

Therefore, we suggested that overall outcomes were generally favorable with targeted management, and permanent deficits were uncommon. These signals do not contradict a putative neuroprotective profile; rather, they emphasize practical risk‐mitigation: (i) adhere to gradual titration and avoid nonregulated compounded products, (ii) anticipate compression neuropathies during rapid weight loss (early bracing/PT; consider decompression if refractory), (iii) monitor for hypoglycemia when dosing is inappropriate or intake is reduced, and (iv) review drug–drug interactions (e.g., statins for myotoxicity risk; valproate where gastric emptying/protein binding shifts exposure).

The broader neuropsychiatric safety of incretin therapies warrants comment. Disproportionality analyses of spontaneous‐reporting databases, including EudraVigilance, have identified psychiatric adverse‐event reports including anxiety, depression, and suicidal ideation for semaglutide, liraglutide, and TZP (Tobaiqy and Elkout 2024). Such pharmacovigilance signals are hypothesis‐generating and susceptible to reporting and media‐attention bias and cannot establish causation or incidence. Reassuringly, a pre‐specified post hoc analysis of the SURMOUNT program (4056 TZP‐treated adults with obesity and no major psychopathology) found no increase in depressive symptoms or suicidal ideation/behavior versus placebo, with a small mean improvement in PHQ‐9 scores and fewer participants shifting to more severe depression categories (Wadden et al. 2026). On current evidence, TZP is not associated with an established causal neuropsychiatric hazard, although monitoring remains prudent, particularly with compounded or nonregulated products.

### 4.6 Implications for Research and Clinical Practice

Tian et al. (2025) used an intraperitoneal (i.p.) dose of 10 nmol/kg in MPTP‐induced subacute PD mice, whereas Yang et al. (2024) administered 10 nmol/kg once‐weekly i.p. in APP/PS1 Alzheimer's disease model mice; in both cases, mouse Km (3) applies (Jacob et al. 2022) (here Km denotes the body‐surface‐area correction factor used in allometric human‐equivalent‐dose (HED) scaling, with Km = 3 for mice and Km = 6 for rats), yielding a HED of approximately 0.004 mg/kg, or 0.28 mg/week for a 70 kg adult. Delvadia et al. (2025) delivered subcutaneous doses of 50 and 100 nmol/kg in rotenone‐induced Parkinson's rats, and Guo et al. (2023) and Hassan et al. (2024) used 1.35 mg/kg either i.p. or s.c. in diabetic and colistin‐treated rats, respectively; for rats (Km 6), the HED converts to about 0.008 mg/kg for 50 nmol/kg (0.56 mg/70 kg) and 0.22 for 1.35 mg/kg (15.4 mg/70 kg), aligning with maximal clinical TZP dosing in humans. Moreover, Misra et al. (2025) utilized 10 nM/kg i.p. in a zebrafish diabetes model. Overall, these values translate preclinical TZP findings to human therapeutic regimens. This places several preclinical doses within a clinically plausible human exposure range, although dose translatability alone does not establish clinical efficacy. These conversion insights clarify that preclinical studies often explore supratherapeutic ranges to establish pharmacological effects and safety, emphasizing the importance of proper dose translation and route selection when planning human studies to maximize relevance and minimize risk.

The current body of evidence regarding TZP demonstrates promising therapeutic potential, as robust preclinical studies in diverse disease models, including PD, Alzheimer's disease, and metabolic dysfunction, suggest efficacy signals in ameliorating disease‐specific phenotypes in these experimental models (with an important negative exception in transgenic Alzheimer's models) via clinically relevant dosing and administration routes. These experimental findings are further supported by real‐world cohort studies, which not only endorse the clinical effectiveness of TZP for weight management and glycemic control but also provide initial reassurance regarding its use across heterogeneous patient populations. However, these findings remain confined to experimental systems, and there is still no direct clinical evidence that TZP slows progression or improves outcomes in Parkinson's or Alzheimer's disease. Nevertheless, documented adverse events linked to TZP include central vertigo and vestibular neuronitis, peroneal neuropathy, psychiatric symptoms, and other unexpected complications, whether due to excessive weight loss, hypoglycemia, or drug–drug interaction. Although the overall incidence remains low relative to the treated population, these reports underscore the necessity for vigilant monitoring of rare but potentially serious side effects, particularly as broader adoption and longer term use may reveal additional safety signals. Taken together, this convergence of positive preclinical outcomes and encouraging real‐world evidence provides a strong rationale for the design and initiation of prospective clinical trials, while emphasizing the importance of robust adverse event surveillance and detailed patient phenotyping to fully elucidate TZP's risk and benefit profile in future studies. This rationale must, however, be tempered by the recent negative Phase 3 trials of GLP‐1RA in Alzheimer's (evoke/evoke+) and PD (Exenatide‐PD3), which caution against assuming that preclinical and observational promise will translate into clinical neuroprotection (Vijiaratnam et al. 2025; Cummings et al. 2026).

The evidence supports several directions for future investigation. First, translational research should bridge mechanistic and clinical domains by integrating biomarker endpoints (neuroimaging, cerebrospinal fluid proteins, and neuroinflammatory markers) into metabolic trials of TZP. Second, dose–response relationships and treatment windows should be systematically tested in diverse neurodegenerative models, including tauopathy and vascular dementia paradigms, to define optimal exposure parameters. Third, observational pharmacoepidemiology could be strengthened through active‐comparator, new‐user designs, and the inclusion of neurological endpoints within existing real‐world evidence frameworks.

Clinically, awareness of weight‐loss–related neuropathies, drug–drug interactions, and iatrogenic hypoglycemia is essential. Structured monitoring of neurological symptoms in patients receiving TZP—especially at higher doses or with rapid weight reduction—may preempt complications. Moreover, interdisciplinary coordination between endocrinologists and neurologists could facilitate early recognition of both beneficial and adverse neurological outcomes.

### 4.7 Strengths, Limitations, and Gaps

The principal strength of this scoping review lies in its comprehensive mapping of mechanistic and clinical domains, integrating diverse evidence streams from molecular to population levels. Validated appraisal tools (SYRCLE, CAMARADE, ToxRTool, NOS, and JBI) ensure transparent quality assessment.

However, this review also exposes significant methodological heterogeneity and reporting limitations across the evidence base. In animal studies, SYRCLE risk‐of‐bias appraisal indicated frequent “unclear risk” ratings for sequence generation, allocation concealment, and blinding, largely due to incomplete methodological descriptions. Sample sizes were modest, outcome measures varied, and dispersion metrics were often unreported, limiting reproducibility and cross‐study comparability. Few studies included structured safety or toxicity assessments, leaving uncertainties about CNS and systemic tolerability at higher or chronic doses. Future preclinical standards should adopt ARRIVE 2.0 and SYRCLE reporting standards, include both sexes, and employ standardized behavioral and molecular endpoints relevant to human translation.

In vitro studies were generally rated “reliable with restrictions,” reflecting adequate internal validity but common omissions such as purity confirmation, solvent controls, and absence of positive comparators. Single concentrations and short exposure windows constrain conclusions about dose–response and chronic effects.

Human retrospective cohorts achieved high NOS scores, indicating strong reporting, but are inherently limited by confounding by indication and exposure misclassification. None incorporated direct neurological outcomes such as cognition, neuroimaging, or fluid biomarkers, and medication adherence was inferred rather than verified. Furthermore, TriNetX‐based datasets may under‐represent frail or uninsured populations, potentially introducing healthy‐user bias.

Case reports satisfied most JBI checklist criteria, providing valuable clinical detail, but are descriptive and non‐generalizable. The absence of systematic pharmacovigilance data precludes estimation of true event frequency. These gaps justify a cautious, descriptive synthesis and highlight the urgent need for standardized preclinical outcome sets, prospective mechanistic trials, and harmonized safety monitoring.

Although there is early evidence that single GLP‐1 RAs may reduce incidence of dementia or mitigate decline and dual GIP/GLP‐1 RAs may have similar effects, no observational or clinical trials have yet tested this for dual agonists. The first clinical trial for TZP in a neurological condition (IIH) is registered recently on ClinicalTrials.gov (Duke University 2025). This scoping review does not aim to determine safety and efficacy of TZP for neurological conditions. Instead, it maps the existing landscape of mechanistic, preclinical, and early clinical evidence relevant to neuroprotection and neurological safety. This helps identify consistent findings, gaps, and what study designs are warranted.

## 5 Conclusion

Current evidence suggests a biologically plausible neuroprotective potential for TZP across cellular and animal systems, but clinical neuroprotection in humans, particularly in Parkinson's and Alzheimer's disease, is still lacking. Observational human cohorts provide preliminary, class‐consistent signals for reduced neurodegenerative and cerebrovascular outcomes, but causal neuroprotection remains unproven. Case reports chiefly describe reversible neurological events linked to dosing practices, drug interactions, or rapid weight loss rather than direct neurotoxicity. Overall, TZP showed hypothesis‐generating neuroprotective signals, for which properly designed mechanistic and early‐phase clinical studies are needed before any claim of disease modification in Parkinson's or Alzheimer's disease can be made. Prospective studies should incorporate pre‐specified neurological endpoints (cognition, imaging, fluid biomarkers, and quantitative vestibular testing), active‐comparator new‐user designs where feasible, and structured safety monitoring that includes hypoglycemia risk, compression neuropathies, and drug–drug interactions. Notably, the recent failure of the largest GLP‐1 receptor agonist trials in Alzheimer's (evoke/evoke+) and PD (Exenatide‐PD3) to demonstrate disease modification tempers expectations for incretin‐based neuroprotection and underscores the need for caution. Among neurological indications, IIH currently represents the most actionable near‐term translational target for TZP, uniting a plausible direct cerebrospinal‐fluid‐reducing mechanism with its established metabolic efficacy.

## Author Contributions

**Yousef Hawas**: conceptualization, methodology, data curation, investigation, validation, visualization, writing – original draft, project administration. **Ahmed Farid Gadelmawla**: writing – original draft. **Yasmin Negida**: writing – review and editing. **Abdallah Khatatbeh**: data curation, validation, investigation. **Dalia Kamal Ewis**: writing – original draft, data curation, investigation, validation. **Mohamed Abouzid**: writing – original draft. **Ahmed Negida**: supervision, writing – review and editing.

## Funding

The authors have nothing to report.

## Ethics Statement

The authors have nothing to report.

## Conflicts of Interest

The authors declare no conflicts of interest.

## Supporting information




**Figure S1**: Summary of the quality assessment of in vivo studies using SYRCLE tool.

**Table S1**: Summary of the quality assessment of cohort studies using NOS checklist.

**Table S2**: Summary of the quality assessment of invitro studies using ToxRTool.

**Supporting Information File 1**: Detailed quality assessment of all included studies expressing the gaps in each domain.

## Data Availability

Data sharing not applicable to this article as no datasets were generated or analyzed during the current study.
