# Supplementary material for: The Neuroprotective Potentials of Dual GIP/GLP1‐RA (Tirzepatide): From Preclinical Experiments to Clinical Trials: A Scoping Review
Source: Brain Behav. 2026 Aug 17;16(8):e71702. doi: 10.1002/brb3.71702 (PMC13482023; doi:10.1002/brb3.71702)
Supplement: Supplementary file 1 [file BRB3-16-e71702-s001.docx]

| **Study ID** | **Selection** | **Comparability** | **Outcome** | **Total score** |
| --- | --- | --- | --- | --- |
| **Lin 2025** | *** | ** | *** | 8 out of 8 |
| **Azzam 2024** | *** | ** | *** | 8 out of 8 |
| **Toraih 2025** | *** | ** | *** | 8 out of 8 |

Table S1: Quality assessment for included cohort studies by New Castle Ottawa Scale.
