# Supplementary material for: The Neuroprotective Potentials of Dual GIP/GLP1‐RA (Tirzepatide): From Preclinical Experiments to Clinical Trials: A Scoping Review
Source: Brain Behav. 2026 Aug 17;16(8):e71702. doi: 10.1002/brb3.71702 (PMC13482023; doi:10.1002/brb3.71702)
Supplement: Supplementary file 2 [file BRB3-16-e71702-s004.docx]

**Table S2:** Quality assessment of In vitro studies by ToxRTool

| **Study ID** | **Criteria Group I: Test substance identification** | **Criteria Group II: Test system characterisation** | **Criteria Group III: Study design description** | **Criteria Group IV: Study results documentation** | **Criteria Group V: Plausibility of study design and data** | Overall total Scores | Final Decision |
| --- | --- | --- | --- | --- | --- | --- | --- |
| **Jiangtao Wang 2025** | 3 | 3 | 4 | 3 | 2 | 15 | Reliable with restrictions |
| **Fontanella 2024** | 3 | 3 | 4 | 3 | 2 | 15 | Reliable with restrictions |
| **Duozi Wang 2025 - In vitro part** | 3 | 3 | 5 | 3 | 2 | 16 | Reliable with restrictions |
| **Yang 2024 - In vitro Part** | 4 | 3 | 5 | 3 | 2 | 17 | Reliable with restrictions |
| **Tian 2025 – In vitor part** | 2 | 3 | 5 | 3 | 2 | 15 | Reliable with restrictions |
